# Voluntary Hospitals and National Insurance

**Published:** 1912-01-20

**Authors:** Thos. Alex. Cook

**Affiliations:** Chairman of the General Committee of Management of West Ham and Eastern General Hospital.


### A SYMPOSIUM OF HOSPITAL WORKERS.

**[We invite correspondence from our readers and all interested in the probable effects of the National Insurance Act on the Voluntary Hospital system. All letters should be accompanied by the name and address of the writer, but not necessarily for publication.]**

### Voluntary Hospitals and National Insurance.

By THE CHAIRMAN OF THE WEST HAM HOSPITAL.

Sir,—In reading your always interesting periodical, I was much impressed with the views set forth or referred to on the above-mentioned subject. I notice with interest that Mr. Sydney Holland believes that no beds will be closed; while others think that they see signs of the already alienated guinea.

I regret to see that some of those responsible for the management of Voluntary Hospitals took advantage of the season of peace and goodwill to protest, and I most sincerely deprecate the expression "blackleg doctors!" Surely every practitioner is entitled to be credited with conscientious motives.

"House Governor's" letter is eminently sensible, courteous, and thoughtful, and seems to me to give an admirable lead to hospital workers as to the profitable mental attitude.

Doubtless a proportion of the guinea givers, from whom the annual extraction is a painful operation, will jump at this opportunity: some of them have already done so, though why, until the "shoe pinches," I fail to see. Some do, every year, on some excuse more or less valid. The increase of death duties; the higher income tax; the licensing legislation; and last, but by no means least, the Budget, have all been hooks on which to hang excuses.

We Voluntary Hospital people are all beggars: many of us trained beggars; and we are hard to resist sometimes. Until the millennium arrives, the hospital secretary or treasurer will always come across those who are or have been awaiting a sufficiently valid excuse to harden their unwilling hearts.

Personally, I look upon the above aspect of the question as one to be faced, but the least cogent. While we are efficient, we shall get money as in the past. So far I agree with "House Governor." And I for one believe that while there is need for healing, the profession—whether they approve of the Bill or not—will help to heal. The great outstanding difficulty (not insurmountable, I hope) is this: Granting "House Governor's" argument that "the Government will have to act its part and make charitable grants to hospitals," what will be the position of the honorary staff? I for one am quite prepared to "wait and see." I know of no body of men who are so keen in charity as the medical men; none so enthusiastic for the efficient working of Voluntary Hospitals: I feel sure that a *via media* will be found, though so far it is a dark road to me and many like me.

Certainly our hospitals will cost, bed for bed and patient for patient, many times more if controlled by State than under the voluntary system. Then I think they will lose in the spirit of hospitality and charity which is so prominent a feature right through; and in losing this I fear they will suffer in healing potentiality.

In conclusion I hope that all interested, being charitable people, will take a broad view like "House Governor," and while endeavouring to obtain the best solution for the hospitals, refrain from strong and inflammatory language more suitable for the hustings than for the Hôtel Dieu.—Yours faithfully,

January 5. Thos. Alex. Cook.

Chairman of the General Committee of Management of West Ham and Eastern General Hospital.

